# Consciousness in Piaget: possibilities of understanding

**DOI:** 10.1186/s41155-018-0110-3

**Published:** 2018-11-15

**Authors:** Tania Stoltz

**Affiliations:** Departamento de Teoria e Fundamentos da Educação-DTFE, Federal University of Paraná, Edifício D. Pedro I rua general carneiro, 460 , Curitiba, 80.060-450 Paraná Brazil

**Keywords:** Consciousness, Grasp of consciousness, Piaget, Causality, Implication, Logical implication

## Abstract

The objective of this article is to analyze the understanding of the concept of consciousness in Piaget. The theme of consciousness is a key concept in Piaget’s theory and he is one of the few psychologists to offer a theoretical structure for the understanding of this theme. Notwithstanding, his proposal for understanding consciousness has been little approached or discussed. Grasp of consciousness, for Piaget, can be understood as the cognitive process of assimilating one’s own functioning or that of the other when interacting with physical objects, people, and oneself. The process of constructing grasp of consciousness is explained by meaningful implication, reflecting abstraction, and equilibration. The dialectics between body and mind and between causality and implication pervade the discussions on consciousness in Piaget’s work. Consensus is not found in the literature with regard to Piaget’s conception of consciousness in virtue of this theme being dealt with at different times in his works with slightly distinct approaches. His works following the 1960s offer new possibilities of understanding the concept of consciousness. Although Piaget moved on in his formulations about consciousness, the essence of his ideas can already be found in his early works.

## Introduction

The theme of consciousness has been gaining renewed space as an object of study both with regard to investigations concerning the third person (in relation to the point of view of the observer, the behavioral or physiological aspect) and also concerning the first person (in relation to the point of view of the subject, the experiential or qualia aspect). (Weger and Wagemann 2015a, 2015b; Weger et al. 2016; Pons et al. 2012; Meyer et al. 2018; Piccinini 2003; Bitbol and Petitmengin 2013, among others). The emergence of new technologies for evaluating cerebral activity has contributed to this renewed interest (Weger and Wagemann 2015a; Schacter et al. 2011) in the theme of consciousness, despite their being based on third person evaluation. The theme of consciousness is a key concept in Piaget’s theory, and he is one of the few psychologists to offer a theoretical structure for the understanding of this theme (Ferrari 2009; Ferrari et al. 2001; Ferreiro 2001; Morgado 1998; Pons and Harris 2001; Pons et al. 2012). Nevertheless, “despite the enduring influence of Piaget’s work in psychology, his conception of consciousness remains relatively unknown. Among the substantive works that synthesize Piaget’s scholarship, his conception of consciousness is never fully presented and discussed” (Pons et al. 2012, p. 96).

As such, this article intends to analyze the understanding of the conception of consciousness in Piaget. The issue of consciousness was always present in Piaget’s works. In *Recherche* (Piaget 1918), the embryon of the idea of the development of stages of consciousness can already be found in the person of the protagonist Sébastien. Piaget (1924/1969, 1936, 1937, 1941/1946) also mention increasing grasp of consciousness of the organizing activity inherent to life itself. The progress of reasoning depends on this grasp of consciousness. This idea is announced more precisely, for the first time, in Introduction to Genetic Epistemology (Piaget 1950) and discussed at a conference relating to problems involving the study of consciousness (Piaget 1954). According to Ferrari (2009), in his main works about consciousness (Piaget 1974a and Piaget 1974b), Piaget increasingly refines his initial ideas and proposes that consciousness involves reconstruction, whereby practical schema is transformed into concepts. “Indeed, it is this ability to explicitly conceptualize logical necessity and how that ability emerged from embodied action in the world that was a guiding idea behind much of Piaget’s research program. Oddly, it is a problem that has not received much attention in the contemporary science of consciousness, which focuses on how experience can be embodied and conceptualized in what Piaget would have considered very concrete ways” (Ferrari 2009, p. 289).

### Grasp of consciousness in Piaget

Grasp of consciousness, in Piaget, relates to a psychological process and to psychological functioning and is, therefore, related to the contents towards which it is directed. Based on Inhelder and Piaget (1979), we know that form and contents are always related: the former relates to structure and the latter relates to procedures. Piaget investigated the development of grasp of consciousness of practical actions and not the subjective nature of conscious experience, as noted in McGinn (1999). Grasp of consciousness, for Piaget, can be understood as the cognitive process of assimilating one’s own functioning or that of the other when interacting with physical objects, people, and oneself.

Grasp of consciousness, in Piaget, involves above all an epistemological issue dealing with the relationships between technique and science and between action and thought. In this sense, Piaget is interested in why and how grasp of consciousness of one’s own action emerges, both with regard to early success of action (Piaget 1974a/1977) and also with regard to the success of actions in stages (Piaget 1974b/1978). Piaget’s interest is coherent with the theory of cognitive development he proposes, the sequence of which starts with practical action, going on to representation before finally reaching operation. From the epistemological point of view, in solidarity with the psychologist, the interiorization of actions lies at the origin of operatory structures, both mathematical and causal, whence its fundamental role. Consciousness is understood here as an active process. Mere exposure to an environment does not promote the development of consciousness. Piaget 1970, 1974a, 1974b of consciousness as a process can already be seen in Baldwin and in Claparède.

According to Piaget, once consciousness of an action is grasped, whereby for this to happen, the action must be interiorized, the possibility of modifying the action arises. Piaget insists on going beyond the view of mere overall illumination in grasping consciousness, to a view of reconstruction. Grasp of consciousness always involves an action being conceptualized and, therefore, always requires reconstruction. Whether grasp of consciousness is a construction or a reconstruction, grasp of consciousness never occurs suddenly and completely, but rather on levels that provide for increasing integration between the interiorization or logico-mathematical movement and the exteriorization or physical-causal movement. Although these two movements are always parallel, to a great extent they take place unconsciously. There is always a delay in grasping consciousness in relation to the early successes of an action.

Pons and Harris (2001) note that Piaget’s hypothesis of practical success in order to achieve conceptual comprehension is largely accepted nowadays, especially when defined in a contemporaneous manner as procedural knowledge (practical success, involving automatic, and implicit procedures; contributes to success via a bottom-up process) whilst declarative knowledge (conceptual comprehension—voluntary and explicit procedures; contributes to comprehension via a top-down process). Notwithstanding, there is disagreement about the functional and developmental relationship between these two types of knowledge: whether procedural and declarative knowledge develop in an autonomous and simultaneous manner (Mandler 1988); whether procedural knowledge precedes the development of declarative knowledge, whereby the latter is functionally dependent on the former (Karmiloff-Smith 1994; Cole et al. 1994); or whether development of declarative knowledge precedes development of procedural knowledge, whereby the latter depends on the former (Harris 1985, 1989, 2000). In the opinion of Mounoud (1995, 1996), procedural knowledge interacts with declarative knowledge right from birth, and this is in agreement with Inhelder and Piaget (1979).

In Piaget (1977a, 1977b), the reason why consciousness is grasped, or its functional reasons, is related to regulations and non-adaptations (1977). Automatic regulations (with corrections of means of acting) are replaced by more active regulations, with deliberate choices, which assumes consciousness. Supplementing Claparède, Piaget refers to the possibility of grasp of consciousness considering adaptation, i.e., late grasp of consciousness, which does not originate from lack of adaptation. Hence, Piaget’s schema pointing to grasp of consciousness as coming from the periphery to the center of the object (C′) and from the center to the subject (C).

In the schema above, the periphery is defined as the phenomenal part of the object that is to be known. Knowledge arises from the interaction between subject and object and there is no consciousness, initially, of the centers of the subject’s action, nor is there knowledge of the intrinsic properties of the specific object that is to be known. “Grasp of consciousness begins at the periphery (goals and results) moving towards the central region of the action when it seeks to reach the action’s inner mechanism: recognition of the means used, reasons for its choice or for its modifications during the experience, etc.” (Piaget 1974a/1977, p. 198). Progress of consciousness may be linked to the difficulties of the action and also to the assimilation process itself (when there is no difficulty or no non-adaptation) and leads beyond grasping of consciousness of the material action; on the plane of reflected action, it leads to a consciousness of the problems to be solved as well as a consciousness of the cognitive means used and no longer the material means used to solve them. It can be said that grasp of consciousness, in Piaget, encompasses the realm of cognition and metacognition (Pons et al. 2012).

Grasp of consciousness occurs through the mental reconstruction of a physical activity through two mechanisms proposed by Piaget (1975, 1977a, 1977b): majorante equilibration and reflecting abstraction. These two mechanisms imply compensatory regulatory processes that become progressively more conscious and efficient and condition the entire future development of a child. Zelazo (2000) makes an interesting distinction between minimal consciousness and recursive consciousness. Whereas practical knowledge or *savoir faire*, which involves minimal consciousness, remains implicit and unarticulated; conceptualization is experienced as an explicit and articulated memory.

According to Ferrari et al. (2001) and Ferrari (2009), despite Piaget (1977a, 1977b) discussing reflecting abstraction, whereby coordination at a lower level is reconstituted at a higher mental level, he continued to struggle with the issue of reconciling the perspective of the first person with the perspective of the third person in the study of consciousness (Varela and Shear 1999). Quoting McGinn (1999) and Searle (1998), Ferrari et al. (2001) they observe that …“it remains a deep mystery how the brain can generate subjectively felt states or qualia, and this is a mystery that Piaget’s explanation has not addressed”. On the other hand, Ferrari et al. (2001) suggest that Piaget’s answer is similar to that of Frege (1956),

In other words, according to Dretske (1995), concepts help explain how one becomes explicitly conscious of qualia (e.g., how one becomes explicitly conscious of the qualitative difference between scarlet and crimson, and not the innumerable other possible shades of red). In fact, Dretske argues that the biological experience of subjective representations is not independent of one ’s conceptualization of external objects. … This is why Dennet (1991) is correct to say that those with an untrained palate simply do not experience wine the way a professional wine taster does Ferrari et al. (2001).

Ferrari (2009) states that although Piaget was considered critical in relation to phenomenology in its original form, his approach is very close to recent efforts regarding the phenomenology of the embodied subject (Vonèche and Stoltz 2007; Vonèche 2008; Müller and Newman 2008). What is original in Piaget, nonetheless, lies in the epistemological problem of how logical necessity emerged through action, as per Ferrari (2009).

Clearly, though, for Piaget (as for Johnson 2007) symbolic and other forms of abstract knowledge begin in embodied action used to imagine creative possibilities by analogy to bodily action (e.g., opening one’s mouth as analogous to opening a box of matches) (see Vonèche 2008, for a detailed presentation of this progression from action to symbolic thought) (Ferrari 2009, p. 297).

As mentioned, when Piaget elaborates on the explanation of how grasp of consciousness occurs, what makes conscious that which was unconscious, he turns to the hypothesis of conceptualization. As such, grasp of consciousness, right from the outset, involves the transition from the assimilation of the object by means of practical schema to the assimilation of the object by means of conceptual schema. Grasp of consciousness always presumes conceptualization because it implies coordinations. And these are produced slowly through reconstructions. Degrees of conceptualization lead to degrees of consciousness and are related to degrees of integration. Cases of cognitive repression or distortion of observed data and repression of the source of conflict make evident a problem of conscious conceptualization. To quote Piaget:

Having incorrectly foreseen an occurrence contrary to a firmly held conviction (that an intermediary should indeed displace itself smoothly in order to transmit a movement, for instance), in the same way the subject contests the unexpected observed data and thinks it can contest the facts just as it foresaw them. Thus, what is interesting about the situation we are discussing now is that, in such cases, the contested observed data is not a physical fact external to the subject, but rather belongs to its own action and is, therefore, known by the subject only in unconscious acts and not in its conscious conceptualization (Piaget 1974a/1977, p.202).

In short, grasp of consciousness appears in all these aspects as a process of conceptualization that, on the plane of semiotization and representation, first reconstructs and then goes beyond that which was acquired on the plane of schemas of action. From this perspective there is, therefore, no difference between the nature of grasp of consciousness of the subject’s own action and knowledge of the sequences external to the subject, whereby both comprise a gradual elaboration of notions based on a fact, whether this fact consists of material aspects of the action performed by the subject, or whether it consists of material aspects of the actions performed between objects (Piaget 1974a/1977, p. 204).

### The three levels of consciousness

In his extensive study of grasp of consciousness, Piaget clearly distinguishes between three levels of consciousness: material action without conceptualization, conceptualization and material action on the same level, and conceptualization guiding material action. The progression of these levels depends on ever-increasing epistemic solidarity between two opposite movements: the exteriorization or physical and causal movement (C′) and the interiorization or logico-mathematical movement (C), which express the circular relationship between subject and objects: with “the subject only learning to know itself through actions with objects and objects only becoming cognizable owing to the progress of actions brought to bear on them.” (Piaget 1974a/1977, p. 211). According to Piaget, any progress in one movement leads to progress in another. Indeed, Piaget refers here to the knowledge of facts and to the correlated knowledge of inferences, in addition to forms of abstraction, namely empirical and reflective abstraction.

**Fig. 1:**
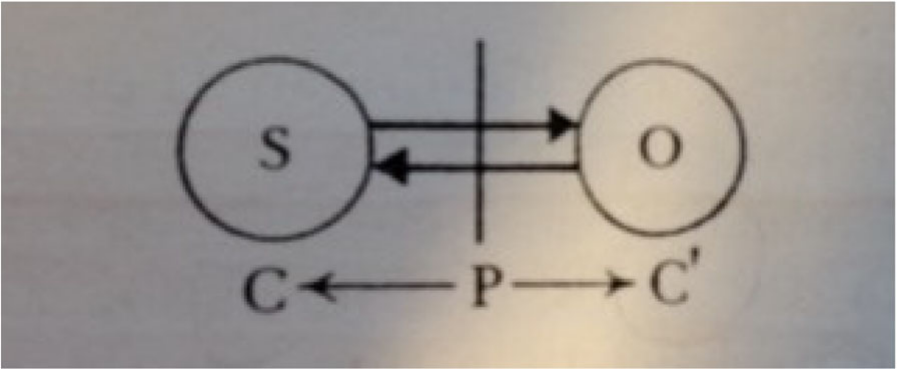
Piaget’s schema of grasp of consciousness. Sources: Piaget 1974a/1977, p. 199

On the level of material action without conceptualization, corresponding to the sensorimotor stage, the process of interiorization enables the construction of a schema logic, prior to language and thought. The exteriorization or physical and causal process, in turn, is characterized as an always greater accommodation of object assimilation schema, culminating in the construction of instrumental conducts (stick, support etc. conduct), spatial-temporal spaces (practical group of displacements), and an objectivated and spatialized causality, following the purely phenomenist forms of the origins in the periphery P (see Fig. 1).

The more a schema comprises links with others, the more flexible it becomes in its applications to objects; but, inversely, the more it multiplies its accommodations, the more these variables favour reciprocal assimilations. (…) group spatial-temporal structures, object permanence, causality spatialization etc., are the result of the coordinations of schema logic, but in these cases they are attributed to objects, in the same way as cinematic and dynamic problems, imposed on subjects by their experience of objects, are fertile incitements in the construction of this logic of actions (Piaget 1974a/1977, p. 210).

On the level of conceptualization, corresponding to the concrete operational stage, the interiorization movement (C) is characterized by a generalized process of grasp of consciousness of one’s own action, which is equivalent to the process of interiorizing material actions through the diverse forms of semiotized representation: language, mental images, drawings, etc. Right from the outset, this process is at one of the extremes of two possible types of abstraction: empirical abstraction and reflecting abstraction. Empirical abstraction enables a kind of description of the data of observed material characteristics of an action; whilst reflecting abstraction takes from the coordinations of the action that which is essential for building inferential coordinations which, on this level of conceptualization, enable the establishment of relationships and the interpretation of observed data, although always interchanging with those of the object. As such, on this level conceptualization, becomes operatory (capable of reasoning and operatory structuring—number, seriation, classification, etc.). But the underlying structures, as well as the very mechanism of reflective abstraction, remain unconscious. The exteriorization or physical and causal movement (C′) on the second level unfolds two analogous processes. Empirical abstraction based on objects enables the abstraction of observed data (facts, specific happenings, functions, relationships that may be repeated, and legality in general). Reflecting abstraction, in turn, which in the C sense is responsible for operatory structuring, enables, as a result, the formation of causal explanations (attribution of operations to objects themselves, which become operators), which involve a deductive interpretation towards objects (C′). However, not only does the attribution of operations to objects remain unconscious for the subject itself, but also the operatory structures as such, in their logico-mathematical inferences, and this indicates solidarity with the interiorization movement. Piaget studied above all the transition of practical success to the conceptual consciousness of the concrete operational stage (Pons et al. 2012).

Finally, on the third level of grasp of consciousness, which corresponds to the formal operational stage and is the level of reflected abstractions, the reflective process of thinking about oneself begins. Reflective abstractions are the conscious product of reflecting abstractions. The interiorization (C) or logico-mathematical movement, on this level, enables the subject to become capable of formulating theory because it begins operating on operations (INRC group, set of parts and combinatory operations, etc.) and does not just have concrete reasoning, even though it is structured logically. The exteriorization (C′) or physical and causal movement, leads it, as a consequence, to the possibility of varying factors in its experimentations and to taking into account diverse models which may possibly explain a phenomenon, comparing them with the control of facts. Solidarity between interiorization and exteriorization movements becomes greater on this level because of increased capacity of abstraction and because of the paradox stated by Piaget: “adaptation to the concrete data of experience depends on the abstract character of the noetic frameworks that enable it to be analyzed and even to be understood” (Piaget 1974a/1977, p. 211).

For Pons and Harris (2001), the correspondence between the three levels of consciousness and the three stages of cognitive functioning (sensorimotor, concrete operational, and formal operational) is questioned by some authors (Mounoud 1993, 1996; Karmiloff-Smith 1994; Zelazo 1999). In this case, the development of reflective consciousness would be closely linked to the particular activities or processes on which it focuses, and this would be maintained throughout development. In this case, some of Piaget’s observations of babies could be indicating early conceptual consciousness, even in the sensorimotor stage. For example, when Jacqueline (Piaget’s daughter), aged 13 months, repeatedly sinks toys in the bathtub to observe how water squirts, throws a toy to see how it floats, or submerges it to see if it will come to the surface. These experiments can be seen, according to Pons and Harris, as indicating early conceptual consciousness even at the sensorimotor stage. “The baby has a conceptual (and not just a practical) consciousness of his or her functioning (and not just of the result) and tries to understand that functioning (and not just to achieve success)” (Pons and Harris 2001, p. 224). In this respect, one can have recourse to Piaget’s observation of the two movements: the interiorization or logico-mathematical movement and the exteriorization or physical and causal movement, which occur at each level of grasp of consciousness, including in the sensorimotor stage. These two parallel movements occur unconsciously and grasp of consciousness always comes after early successes of action, according to Piaget’s explanation. For Piaget, grasps of consciousness in the sensorimotor stage are more superficial and linked to practical schemas.

### Meaningful implication, past, and future in the grasp of consciousness

Piaget emphasizes that the most general characteristics of the conscious states, right from the most elementary grasps of consciousness, is “meaningful implication”, which is characterized by expressing significations and bringing them together in the form of a connection. By means of meaningful representations and making use of semiotic instruments (language, images, drawings, etc.), it is possible to translate what relates to action and to its contexts, but the key functional point of the coordinations themselves is the operational coordinations system because it transforms the objects of thought, like action modifies material objects. As such, it is Piaget’s (1974b/1978) understanding that operation is not a representation of action, it continues to be action, since it builds novelties, but it becomes meaningful action and no longer physical action, because the means it uses are of an implicative nature and no longer of a causal nature.

Piaget argues in favour of isomorphism of causality and implication in grasp of consciousness. Implication is a connection between significations, “if the causal coordinations of actions enable their material goals to be achieved, in an acquisition that comprises their value, albeit a limited acquisition, the system of meaningful implications provides an element that is not understood, neither with regard to the goals, nor with regard to the means used: it is the determination of the reasons, without which successes represent only facts without meaning” (Piaget 1974b/1978, p. 179). Piaget then asks himself how reasoning becomes autonomous, given that there is isomorphism between the causal structures of actions and their objects and the implicative structures of thought. The answer considers two points: by exploring the reasons for a physical phenomenon, real relationships observed can be situated on a plane of possible relationships, and this demands going beyond action. On the other hand, on the third level of the development of consciousness, when conceptualization guides action, the subject’s operational power is prolonged indefinitely, given that the subject can build operations on top of operations, which necessarily go beyond the limits of action. “In short, the understanding of reason or the quest for it can only go beyond practical successes and enrich thought to the extent that, for the two preceding and conjoint motives, the world of “reasons” grows beyond that which is possible and thus extends beyond that which is real” (Piaget 1974b/1978, p.179).

An important proviso made by Piaget refers to going beyond action through the use of reason. If conceptualization goes beyond action or reason goes beyond the success of action, enabling an unlimited number of new operations beyond previous operations, “this does not mean that there are pure constructions in this that are not referenced to a retrospective movement which leads once more from the periphery to the centre of operational structures. It is clear that, on the contrary, each new construction is supported, at its starting point, by elements that are taken from previous levels by abstractions through reflections” (Piaget 1974b/1978, p. 180). On the other hand, in the movement towards exteriorization or the process of causal explanation, an indefinite alternation can also be found in relation to the whys and wherefores of the models found and which are close to an object, even on the higher levels of scientific thought. In short, right from a child’s experimental actions, there is constant equilibrium between movements of interiorization (relating to the construction of operational structures) and movements of exteriorization (relating to the construction of causal explanations).

Grasp of consciousness, for Piaget (1974b/1978), involves an evolution that is always oriented and does not imply a future action on the present. It involves, therefore, a direction at each stage that oscillates between the determination of the past and being infinitely open to unforeseeable novelties. It is through the construction of deductive instruments in one stage that a new and unforeseen construction can retrospectively appear as being necessary. In this sense, goals are unfolded by equilibration, by the process itself. It is equilibration that precedes regulations.

Therefore, an essential factor of grasps of consciousness are situations of disequilibrium and re-equilibration, which involve the role of conflicts and contradictions in the process of majorante equilibration (Piaget 1975). Initial disequilibrium in grasp of consciousness is related to the primacy of the positive affirmations or characteristics of the actions to be performed, as well as the primacy of situations, as opposed to denials, diminutions, or negative characteristics. Although positive elements are always countered as a logical necessity by corresponding negative elements, little value is given to negative elements at the initial levels. Positive affirmations or aspects are at the periphery of an individual’s activities, since the positive aspects of that which can be observed are perceived before its negative aspects. Denials refer to the more central regions of action. The omission of negative elements provokes an entire series of disequilibrium and contradiction in the process of grasping consciousness.

### Physical and psychological dimension of grasp of consciousness

Ferrari et al. (2001) note that understanding consciousness in Piaget requires an understanding of how each subject acquires consciousness and how the physical and cognitive systems are interrelated. “For Piaget, then, understanding consciousness involves understanding both how the individual subject acquires necessary knowledge of abstract and physical objects and how the cognitive and physical systems involved in generating such knowledge relate to each other” (Ferrari 2009, p. 289). In this sense, Piaget’s proposal of consciousness necessarily takes into consideration (Ferrari et al. 2001): (1) the relationship between subject and object, which will lead to the development of conceptual knowledge of objects and (2) the relationship between cognitive activity and neural activity, with regard to the relationships between psychological representations and neurobiology.

As for the relationship between subject and object, Ferrari et al. (2001) and Ferrari (2009) consider that all discussion of reality evokes the traditional subject-object dichotomy, which at times privileges the subject (idealism) or the object (realism), or the relationship between the two: interactionism. According to these authors, Piaget’s position is directed towards internal interactionism, as the original synthesis of idealism and realism. This interactionism considers that the world exists prior to the subject’s knowledge, but that the subject only gets to know it by acting on it. This fourth solution (Piaget 1950), according to Ferrari et al. (2001), concerns the relationship between mathematical thought and reality and considers mathematical relationships not just in the subject (apriorism) nor just in the object (empiricism), nor in an interaction between subject and an object external to it (external interactionism), but rather to an interaction between both of them which remains internal to the subject itself. If objects and physical reality were different, mathematics and logic would be different because in a different world, mental and physiological structures would be distinct, and life itself would have emerged from a physical and chemical structure different to ours. It is within the subject, to the extent that the subject unfolds its functioning of reality from its biological and physicochemical roots, that the subject is in interaction with the object with regard to the general coordination of its acts. It is for this reason that the coordinations are also in agreement with reality, from which they take their source. “Internal interactionism (the fourth solution) remains an elegant approach to the subject-object problem. It suggests that becoming conscious- a conceptualization- is ‘embedded within and bound up with practical activities’ necessary for the emergence of conscious meaning” (Ferrari et al. 2001, p. 200, citing Müller 1999, p. 13). It is our experience of developing in the world that determines our understanding of external reality. Notwithstanding, Piaget was always concerned with the question of the emergence of the logical necessity (always true) of a series of specific actions (only empirically true). In this sense, Piaget can be considered to be much more of a constructivist realist (Neuhäuser 2010), or an objective idealist (Kesselring 1999), but not a radical constructivist (Rusch and Schmidt 1994).

Ferreiro (2001) recalls that the expression internal constructivism was used by Piaget (1950) in relation to mathematical knowledge, but disagrees with the use of this expression to characterize Piaget’s later works. What Piaget emphasized, above all following the studies on causality (from the late 1960s to the early 1970s), is the interactionism inherent to constructivism. For Ferreiro (2001), Piaget modified previous formulations. “Beginning with the studies on causality, Piaget faced the necessity of constructing new interpretative schemes that finally led him to a new equilibration stage of his own theoretical development” (p.215).

The second point approached in the article by Ferrari et al. (2001), relating to Piaget’s approach to consciousness, pertains to the non-reductionist interpretation of the relationship between psychology and neurobiology in a Piagetian discussion on consciousness. Whereas physical causality is associated with the physiology of consciousness, logical implication is related to the psychology of consciousness and involves non-mechanistic logic. “A *entails* or *implies* the conscious phenomenon B by necessity, constraint, or consequence—a connection that thus exists intentionally and subjectively within the individual mind. This level of explanation concerns the phenomenon’s *raison d’être* and thus becomes a logical implication by showing why phenomenon B is what it is” (Ferrari et al. 2001, p. 202). Piaget and Garcia (1987) go even further in this direction by analyzing how children develop intentional logic based on implications, as opposed to extensional logic. At the end of his life, Piaget (1977a, 1977b; Piaget and Garcia 1983, 1987) began reformulating his vision of the relationships between biology and psychology based on Prigogine’s theory of open systems, including the development of knowledge about the world and one’s own conscious mind based on principles of self-organization. Causal explanations and implications progressively influence each other through an increasingly perfect correspondence between causal connection, inherent to physical explanation, and implicative connection, inherent to psychological analysis (Piaget and Garcia 1971; Stoltz 2005).

For Piaget (1970), equilibration provides an example in which isomorphism was almost complete; (…) How does one go from biological rhythm or regulation to cognitive operations that establish norms? Vonèche (2008) suggests that Piaget believed in a rule-seeking capacity of the human mind, and that every biological (and by extension cognitive) system tends to optimize its equilibration, which is by definition immanent to it. But why? Piaget does not say. Such activity is perhaps what Taylor (1989) would call a hypergood that cannot itself be questioned (Ferrari 2009, p. 298).

To the extent that the physiological and the psychological explain the same actions, at times the respective levels of explanation are too closely related and result in an integrative monism, to be perceived through the future progress of science. According to Ferrari et al. (2001) and Ferrari (2009), this suggests an integration between conscious processes and neuroscience. For Piaget (1950), reciprocal assimilation between mind (spirit) and body will lead to simultaneous understanding of the relationships between mind (spirit) and body and of the relationships between subject and object. The idea of an integrative monism is explained by the integration of the dualism between causality and implication. Coexistence between monism and dualism can be understood based on complex systems, this being a perspective to which Piaget became closer at the end of his work with physicist Rolando Garcia (Piaget and Garcia 1971, 1983, 1987).

In a criticism of Ferrari et al. (2001), Ferreiro (2001) expresses the impossibility of admitting “two types of causality” in the works of Piaget, whereby one would be irreducible in relation to the other. Ferrari et al. (2001) observe that the new studies on the theme of causalities in the 1960s lead Piaget to identify objects as operators, refining the understanding of the object pole in his constructivist viewpoint. According to Ferreiro, Piaget insisted on the distinction between causal relationships (leading to empirical laws) and causal explanations (involving the idea of need, which refers to logic) in the relationships between causes and effects.

Logico-mathematical structures are the result (through reflexive abstraction) of the general coordination of actions, and these actions, from the very beginning, are actions of an organism related to external entities of their environment (well before there were actions of a subject on external objects). The logical implications that will be attributed to the objects themselves have their source in the elementary anticipations originated by the coordinations of actions. Piaget insisted on this point in his last years (*Toward a Logic of Meanings*, 1987/1991: ‘Logical relations are constructed at the same time that the empirical world is being organized, and they are an inherent part of the organizing process’ (p.27) (Ferreiro 2001, p.216).

The correspondence between the causal transformation of objects and operatory transformation of the subject is due to the fact that self-action is both dependent on the physical laws of the object in general and also the source of the subject’s operations. This situation points to a new way of conceiving a scientific explanation. After studying causality, Piaget notes that objects themselves need to be conceived of as operators. Piaget’s main works on consciousness (Piaget 1974a, 1974b), written after his studies on causality, point to the relationship between causality and grasp of consciousness. The scientific explanation of consciousness would, therefore, not lie in the association of ideas or in the innate sequence of structures, but rather is a constructivist interactionist explanation.

## Conclusion

The intention of this article was to analyze the understanding of Piaget’s concept of consciousness. There is no consensus as to what consciousness is for Piaget owing to this theme being dealt with at different moments of his work and with slightly differentiated approaches. On the other hand, Piaget’s studies on causality, conducted during the 1960s, have been little explored in scientific discussion, in the same way as his works following this period, especially the two works dedicated to the study of consciousness (Piaget 1974a, 1974b) which were prior to the proposal of the new model of equilibration in 1975. The third equilibration model that points to the logic of significations, arising through a partnership between Piaget and Garcia, as well as the second equilibration model, have implications for the understanding of consciousness based on Piaget, because they describe with greater precision the role of the object as an operator acting on the subject and because of the constructivist viewpoint on which it is based. Piaget’s works from the 1960s onwards need to be better known, since they open the way to infinite possibilities of construction based on the ideas of complex systems. This is also the case for studies involving grasp on the consciousness of oneself and of others, integrating cognitive, social, affective, and moral aspects. Stoltz (2010) and Stoltz et al. (2014a, 2014b) are cited here as initiatives in this direction. On the other hand, interesting discussions involving the concept of grasp of consciousness can be seen in Champlain et al. (2018); Cooper and Stoltz (2018); Becker (2017); Montoya (2017); Othman and Stoltz (2017); Pinheiro and Becker (2014); Stoltz et al. (2014); Carneiro et al. (2015) and Moro (2005).

In Piaget, the process of constructing grasp of consciousness is explained by meaningful implication, reflecting abstraction, and equilibration. Although Piaget moved on with his formulations regarding consciousness, the essence of his ideas can already be found in his first works. It can be said that Piaget refined concepts such as equilibrium and disequilibrium between nature and spirit, grasp of consciousness, and reflection, which are already present in his first works. It can be said that there is a quest for a synthesis between the first and the third person in Piaget’s studies of consciousness because he refers to the conscious knowledge that the subject has of its own functioning and of its object of knowledge. On the other hand, Piaget assimilated the problem of consciousness into his own scheme of cognitive development, making it virtually equivalent to verbal reflections on actions. In this way, he came closer to Freud and William James with regard to the conception of consciousness and conscious products.

## Authors’ information

TS received a scholarship from the Fondation Jean Piaget pour recherches psychologiques et épistémologiques (Geneva) for the conclusion of her doctorate and undertook her postdoctoral research at Jean Piaget Archives on the subject of *causal explanation*, *grasp of consciousness*, *and social interaction.*
